# Spontaneous Expulsion of a Foreign Body from the Bronchi

**Published:** 1891-01-24

**Authors:** 


### SPONTANEOUS EXPULSION OF A FOREIGN BODY FROM THE BRONCHI.

Dr. Keferstein, of Alt Döbern, was called to see a woman, aged 58, the wife of a day labourer, who a month before choked while eating some soup made with crushed bones, millet, &c., and was certain that "something had gone down the wrong way." From that time she had suffered from paroxysms of cough, resembling whooping cough or the "tussis convulsiva" of children, coming on at intervals of two or three hours by day and night alike. On one occasion, when lifting a sack of potatoes she had just dug in the field, the paroxysm was so severe that she fell with the load to the ground and thought she would have died. She pointed to a spot on one side of the middle of the sternum as the place she could feel it, and implored Dr. Kerferstein to cut down upon it. He, however, persuaded her to wait a little, and gave her a mixture of full doses of ammonia and senega every two hours. Two days afterwards, at the beginning of a paroxysm she felt something fly into her mouth and spat out a piece of bone 8mm. by 5mm. by 4mm (or $\frac{3}{8} \times \frac{1}{2} \times \frac{1}{3}$ in.) with no sharp angles or stain of blood. Dr. Keferstein having found nothing so useful in aiding expectoration and relieving convulsive cough as senega and ammonia, believes that the expulsion of this body was mainly brought about by the medical treatment.

